# Molecular Cloning and Bioinformatics Analysis of a New Plasma Membrane Na^**+**^/H^**+**^ Antiporter Gene from the Halophyte *Kosteletzkya virginica*

**DOI:** 10.1155/2014/141675

**Published:** 2014-06-30

**Authors:** Hongyan Wang, Xiaoli Tang, Chuyang Shao, Hongbo Shao, Honglei Wang

**Affiliations:** ^1^Key Laboratory of Coastal Biology & Bioresources Utilization, Yantai Institute of Coastal Zone Research (YIC), Chinese Academy of Sciences (CAS), Yantai 264003, China; ^2^Yantai Academy of China Agricultural University, Yantai 264670, China; ^3^University of Chinese Academy of Sciences, Beijing 100049, China; ^4^College of Life Sciences, Shandong Agricultural University, Taian 271018, China; ^5^Institute for Life Sciences, Qingdao University of Science & Technology (QUST), Qingdao 266042, China

## Abstract

A new plasma membrane Na^+^/H^+^ antiporter gene (named as *KvSOS1*) was cloned from the halophyte *Kosteletzkya virginica* by reverse-transcription-polymerase chain reaction (RT-PCR) and rapid amplification of cDNA ends (RACE) technology, which is a homologue of SOS1 (salt overly sensitive 1). The full-length cDNA is 3850 bp and contains an open reading frame (ORF) encoding a protein of 1147 amino acids with a molecular weight of 127.56 kDa and a theoretical pI of 6.18. Bioinformatics analysis indicated that the deduced protein appears to be a transmembrane protein with 12 transmembrane domains at the N-terminal region and a long hydrophilic tail in cytoplasm at its C-terminal region and shares 72–82% identity at the peptide level with other plant plasma membrane Na^+^/H^+^ antiporters.

## 1. Introduction

The salinization of soil has become a widespread environmental problem and an important factor in limiting agricultural productivity worldwide. At present, more than 800 million hectares land in the world is affected by salinity and this amount accounts for more than 6% of the world's total land area [1]. Even worse, the saline soil is still rapidly expanding due to irrigation, improper drainage, entry of seawater in coastal areas, and salt accumulation in arid and semiarid regions [2, 3]. So there is an urgent need to develop salt-tolerant crops which can grow in saline environments to overcome farmland salinization as well as enable agriculture in marginal lands [4–6].

As far as we know, the detrimental effects of salt stress on plant can be summarized into three main aspects. Firstly, saline soil leads to osmotic stress, which makes plants hard to take up water from the soil. Secondly, salt stress may induce ionic toxicity. The increase of Na^+^ and Cl^−^ concentration in the cytosol can negatively affect enzymes and lipids in the cells. When Na^+^ and Cl^−^ concentrations increase to the toxic threshold, cells tend to die. At last, high soil salt concentration can also induce oxidative stress, which can cause a series of oxidative damage [1]. Fortunately, salt-tolerant plants have evolved some special mechanisms of salt tolerance which are to minimize the accumulation of toxic ions in plant tissue, partition them in the apoplast and vacuole, increase the synthesis of osmotic adjustment substances such as proline and betaine for maintaining tissue water status, and enhance antioxidant capacity to prevent the occurrence of oxidative stress [7]. Based on the above physiological mechanism, researchers have successively cloned many genes related to salt stress in various plants (e.g., genes encoding ion transporters, osmolytes, antioxidant enzymes, components of calcium signaling, and others) [8]. Among them, Na^+^/H^+^ antiporter genes have been proved to play an important role in salt tolerance, which are considered as promising genes for breeding salt-tolerant crops via genetic engineering. Under salinity, the great problem faced by plants is to maintain Na^+^ homeostasis in the cytosol because low cytosolic Na^+^ is crucial for cell metabolism [9–11], and this can be achieved by Na^+^/H^+^ antiporters located in vacuolar membrane and plasma membrane. The vacuolar Na^+^/H^+^ antiporters (Na^+^/H^+^ exchangers, NHXs) can actively transport excessive Na^+^ into the vacuole for Na^+^ compartmentation, while the plasma membrane-located Na^+^/H^+^ antiporters are responsible for Na^+^ exclusion from the cytosol to the external medium [12, 13]. Since studies on Arabidopsis showed that overexpression of either the vacuolar membrane Na^+^/H^+^ antiporter AtNHX1 or the plasma membrane Na^+^/H^+^ antiporter AtSOS1 could improve the salt tolerance of transgenic plants [14, 15], more and more studies have focused on the cloning and function of Na^+^/H^+^ antiporter genes from other plant species [16–23]. In the future, more attention should be paid to develop Na^+^/H^+^ antiporter genes and other salt tolerance genes from halophytes because of their inherent and excellent salt resistance.

*Kosteletzkya virginica* (L.), also commonly known as seashore mallow, is a perennial facultative halophytic species in the Malvaceae family, natively distributing in coastal areas containing 0.3 to 2.5% sodium salt (mainly NaCl) from Long Island along the Atlantic coast of the U.S. west to eastern Texas and is also found in coastal areas of Eurasia [24–26]. Because of its economic values and the tolerance to saline soils, this species has been introduced in China and recommended as a potential cash crop for alternative saline agriculture [10, 27]. Cloning some crucial salt stress response genes from such halophyte sources and investigating their characterizations and functions should be valuable for further understanding the molecular mechanism of plant salt tolerance and also helpful for breeding salt-tolerant crops. So this work aimed to isolate a new plasma membrane Na^+^/H^+^ antiporter gene from* Kosteletzkya virginica* and investigate its characterizations, which might not only help to understand the salt tolerance mechanism but also provide valuable genes related to salt tolerance for molecular breeding of salt-tolerant crops.

## 2. Materials and Methods

### 2.1. Plant Materials and Growth Conditions

The seeds of* Kosteletzkya virginica* were collected from Yellow River Delta, Shandong Province, China. The seeds were soaked in concentrated sulfuric acid for 20 min to remove the hard shell and then thoroughly rinsed with deionized water. Subsequently, the processed seeds were sown in plastic flowerpots (with drain holes in bottom) containing washed sand and grown in the artificial climatic chambers (Huier, China), which was controlled under 28/25°C (day/night) with a daily photoperiod of 14 h and relative air humidity of 65%. Seedlings were sufficiently watered with 1/2 Hoagland nutrient solution every 3 days. Salt treatments were conducted by adding NaCl to 1/2 Hoagland nutrient solution. For the isolation of Na^+^/H^+^ antiporter gene, 3-week-old seedlings were treated by 200 mM NaCl for 24 h, and their roots were carefully removed from sands, washed with deionized water, and then harvested. The samples were rapidly frozen in liquid nitrogen and stored at –80°C for the next experiments.

### 2.2. Cloning of* KvSOS1* cDNA by RT-PCR and RACE

Total RNA was extracted from the above mentioned roots using RNAiso Plus (TaKaRa, Japan) according to the manufacturer's instruction. Quality and quantity of total RNA were measured by using a NanoDrop-2000c spectrophotometer (Thermo Fisher Scientific, USA). The first-strand cDNA was synthesized according to the instruction of TransScript All-in-One First-Strand cDNA Synthesis SuperMix for PCR (Transgen, China). Based on sequence alignments of the conserved regions of reported SOS1 genes from various plant sources (*Theobroma cacao*, EOY01238.1;* Populus trichocarpa*, XP_002315837.2;* Ricinus communis*, XP_002521897.1;* Bruguiera gymnorrhiza*, ADK91080.1), a set of degenerate primers (DP-F, DP-R; sequences given in Table 1) were designed and used for the amplification of core fragment of SOS1 from* Kosteletzkya virginica.* The first strand cDNA was used as the template for PCR amplification under the following conditions: 95°C for 5 min, 35 cycles of 95°C for 40 s, 56°C for 30 s, 72°C for 2 min, and 72°C for 10 min. The amplified fragment was ligated into the pGEM-T easy vector (Promega, USA) and sequenced. After the fragment was confirmed to be part of* KvSOS1 *gene by NCBI blast, the 5′ and 3′ ends of the full-length cDNA were further amplified according to the instruction of SMART RACE cDNA Amplification Kit (Clotech, USA). Gene specific primers and nested primers were designed according to the core cDNA sequence. They are as follows: 5′-GSP, 5′-NGSP, 3′-GSP, and 3′-NGSP, as shown in Table 1. The nested PCR was performed in 5′ and 3′ RACE. The PCR products were separated by 1% agarose gel electrophoresis. DNA from the target band was excised from the gels and purified, then ligated into the pGEM-T easy vector (Promega, USA) and sequenced. Finally, the above obtained sequences were spliced and assembled into the full-length cDNA, which was designated as* KvSOS1.*

## 3. Results and Discussion

### 3.1. Cloning and Characterization of the* KvSOS1* cDNA

The full-length cDNA of* KvSOS1* (GenBank accession: KJ577576) was obtained by RT-PCR and RACE methods (specified in Materials and Methods). As shown in Supplementary Figure 1in Supplementary Material available online at http://dx.doi.org/10.1155/2014/141675, the full-length cDNA is 3850 bp, consisting of 5′-untranslated region of 93 bp, an uninterrupted open reading frame (ORF) of 3444 bp, and 3′-untranslated region of 313 bp. The predicted ORF of* KvSOS1 *encodes a protein of 1147 amino acids with a molecular weight of 127.56 kDa and a theoretical pI of 6.18.

### 3.2. Bioinformatics Analysis of* KvSOS1*

**Figure 1:**
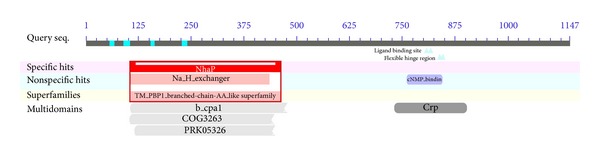
Analysis of conserved domains in KvSOS1.

**Figure 2:**
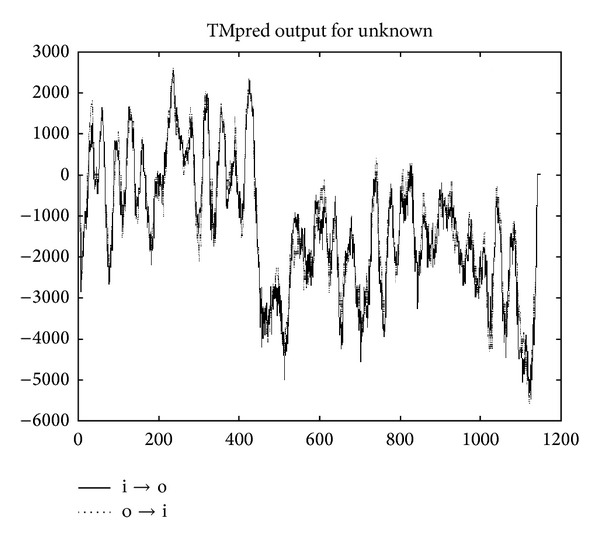
Plot of hydrophobic and hydrophilic areas in KvSOS1.

Conserved domain analysis using CDD of NCBI revealed that the putative protein belongs to the sodium/hydrogen exchanger family (Figure 1), which generally contains 10–12 transmembrane regions at the amino-terminus and a large cytoplasmic region at the carboxyl terminus. Hydropathy plot analysis using the TMpred program further indicated that the obtained* KvSOS1 *encodes a predicted transmembrane protein. The N-terminal region includes 12 predicted transmembrane domains, while its C-terminal region has a long hydrophilic tail in cytoplasm (Figure 2). This is consistent with previously reported for plant plasma membrane NHAs [28–30].

**Figure 3:**
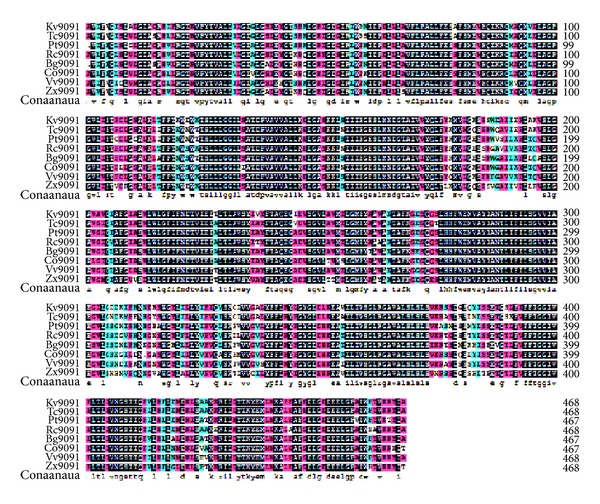
Alignment of KvSOS1 with other SOS1 homologues derived from* Theobroma cacao* (TcSOS1, EOY01238.1),* Populus trichocarpa *(PtSOS1, XP_002315837.2),* Ricinus communis *(RcSOS1, XP_002521897.1),* Bruguiera gymnorrhiza *(BgSOS1, ADK91080.1),* Cucumis sativus *(CsSOS1, XP_004150155.1),* Vitis vinifera* (VvSOS1, NP_001268140.1), and* Zygophyllum xanthoxylum *(ZxSOS1, ACZ57357.1). Identical peptides highlighted in black.

Multiple sequence alignments demonstrated that the deduced amino acid sequence of KvSOS1 is 82%, 74%, 73%, 73%, and 72% identical to those homologues from* Theobroma cacao*,* Populus trichocarpa*,* Citrus sinensis*,* Ricinus communis*, and* Populus euphratica*, respectively, which are all plasma-type Na^+^/H^+^ exchangers. The highest degree of sequence similarity, especially, locates in the transmembrane regions, where it reaches almost 88% between KvSOS1 and TcSOS1 (Figure 3).

**Figure 4:**
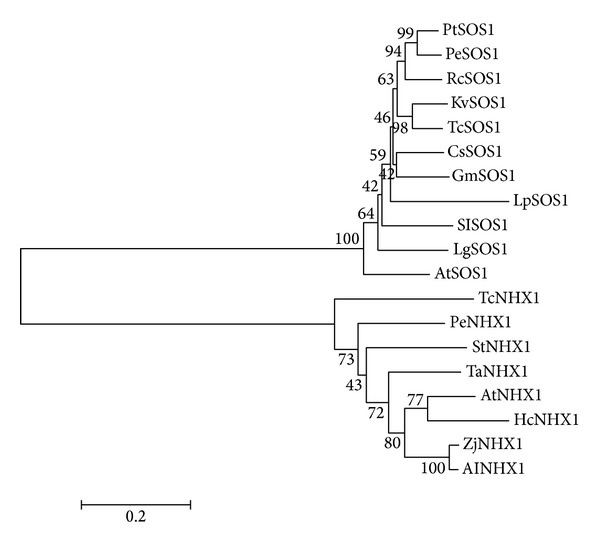
Phylogeny of KvSOS1 and other Na^+^/H^+^antiporter proteins derived from* Populus trichocarpa *(PtSOS1, XP_002315837.2),* Populus euphratica* (PeSOS1, ABF60872.1),* Ricinus communis *(RcSOS1, XP_002521897.1),* Theobroma cacao *(TcSOS1, EOY01238.1),* Citrus sinensis* (CsSOS1, XP_006492282.1),* Glycine max* (GmSOS1, AFD64746.1),* Lolium perenne* (LpSOS1, AAY42598.1),* Solanum lycopersicum* (SlSOS1, NP_001234698.1),* Limonium gmelinii* (LgSOS1, ACF05808.1),* Arabidopsis thaliana *(AtSOS1, AF256224_1),* Theobroma cacao *(TcNHX1, XP_007030791.1),* Populus euphratica *(PeNHX1, ACZ05630.1),* Solanum torvum *(StNHX1, AEN04067.1),* Triticum aestivum* (TaNHX1, AAS17949.1),* Arabidopsis thaliana *(AtNHX1, NP_198067.1),* Halostachys caspica *(HcNHX1, ADK62565.1),* Zoysia japonica* (ZjNHX1, ABY19311.2), and* Aeluropus littoralis* (AlNHX1, AAV80466.1).

**Figure 5:**
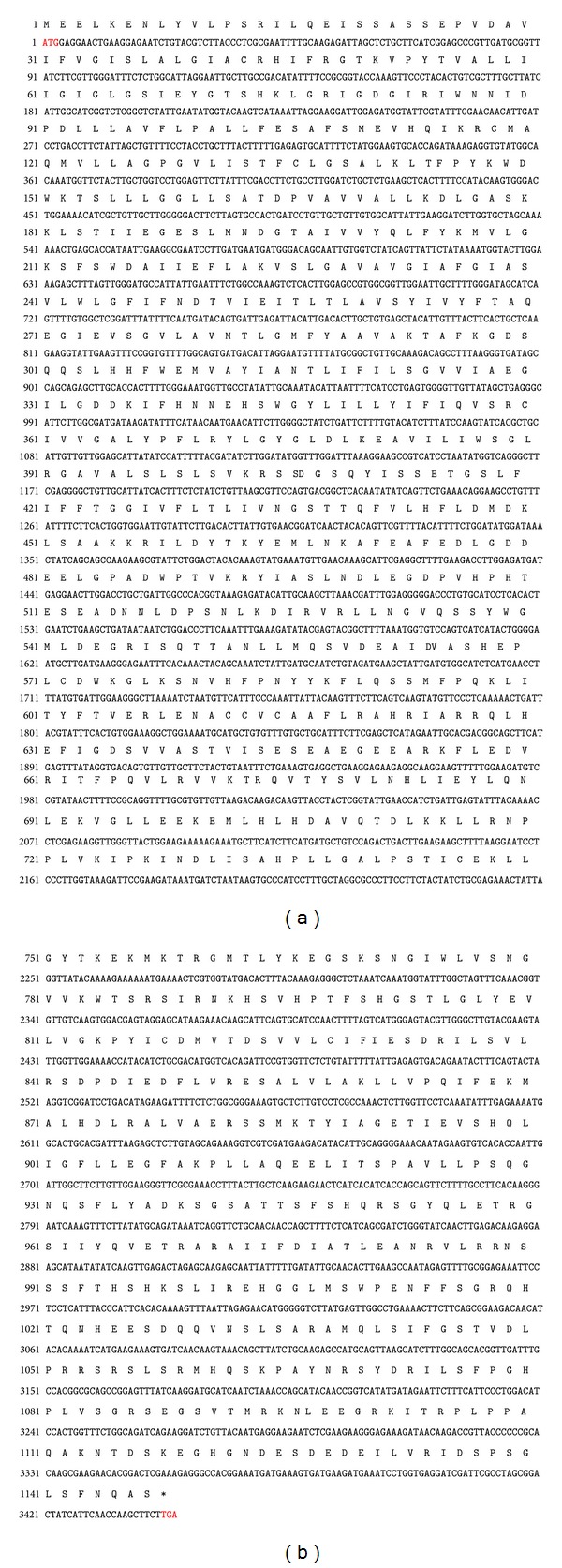
The ORF nucleotide sequence and the deduced peptide sequence of* KvSOS1*. Start codon and termination codon are highlighted in red.

Phylogenetic analysis of some Na^+^/H^+^ antiporters from various plants showed that KvSOS1 formed a cluster with other plant plasma membrane SOS1 homologues and is most closely related to the* Theobroma cacao* homologue (GenBank accession EOY01238.1), which was different from the cluster of plant vacuolar NHX1 homologues (Figures 4 and 5). All these results implied that the obtained* KvSOS1* is a plasma membrane type Na ^+^/H^+^ antiporter gene.

## 4. Conclusion 

*Kosteletzkya virginica* has been proved to be a promising halophyte and has been introduced in China and recommended as a potential cash crop for alternative saline agriculture. In addition, some crucial salt stress response genes can be cloned from it for molecular breeding of salt-tolerant crops. In our study, the full-length cDNA of KvSOS1 was isolated from* Kosteletzkya virginica*. Bioinformatic analysis predicted that it encodes a putative plasma membrane Na^+^/H^+^ antiporter, which has the typical characteristics of other homologous genes. In order to investigate its characterization and function in salt tolerance, the further study would focus on the expression pattern and genetic transformation of* KvSOS1*, which might provide guidance for molecular breeding of salt-tolerant crops.

## Supplementary Material

Supplementary Figure 1: The ORF nucleotide sequence and the deduced peptide sequence of KvSOS1. Start codon and termination codon highlighted in red.

## Figures and Tables

**Table 1:** The primers used in this study.

Primer	Sequence (5′-3′)
DP-F	5′-GG(A/G)GAATCCTT(A/G)ATGAA(C/T)GATGGGAC-3′
DP-R	5′-C(T/C)A(G/A/T)AGC(G/A)CTTTCCTGCCA(C/T)AG-3′
5′-GSP	5′-GCTATCCCAAAAGCAATTCCAACCGC-3′
5′-NGSP	5′-CCAAGTGAGACTTTGGCCAGAAATT-3′
3′-GSP	5′-GTGCATCCAACTTTTAGTCATGGGAG-3′
3′-NGSP	5′-GTGACAGAATACTTTCAGTACTAAGGTC-3′

## References

[1] Yan K, Shao H, Shao C (2013). Physiological adaptive mechanisms of plants grown in saline soil and implications for sustainable saline agriculture in coastal zone. *Acta Physiologiae Plantarum*.

[2] Ashraf M (2009). Biotechnological approach of improving plant salt tolerance using antioxidants as markers. *Biotechnology Advances*.

[3] Tang X, Mu X, Shao H, Wang H, Brestic M (2014). Global plant-responding mechanisms to salt stress: physiological and molecular levels and implications in biotechnology. *Critical Reviews in Biotechnology*.

[4] Chen P, Yan K, Shao H, Zhao S (2013). Physiological mechanisms for high salt Tolerance in Wild Soybean (Glycine soja) from yellow river Delta, China: photosynthesis, osmotic regulation, ion flux and antioxidant capacity. *PloS one*.

[5] Wu X, Zhang H, Li G, Liu X, Qin P (2012). Ameliorative effect of castor bean (*Ricinus communis* L.) planting on physico-chemical and biological properties of seashore saline soil. *Ecological Engineering*.

[6] Li Z, Li G, Qin P (2010). The prediction of ecological potential for developing salt-tolerant oil plants on coastal saline land in Sheyang Saltern, China. *Ecological Engineering*.

[7] Zhang G, Su Q, An L, Wu S (2008). Characterization and expression of a vacuolar Na^+^/H^+^ antiporter gene from the monocot halophyte *Aeluropus littoralis*. *Plant Physiology and Biochemistry*.

[8] Agarwal PK, Gupta K, Jha B (2010). Molecular characterization of the *Salicornia brachiata* SbMAPKK gene and its expression by abiotic stress. *Molecular Biology Reports*.

[9] Maathuis FJM, Amtmann A (1999). K^+^ nutrition and Na^+^ toxicity: the basis of cellular K^+^/Na^+^ ratios. *Annals of Botany*.

[10] Tester M, Davenport R (2003). Na^+^ tolerance and Na^+^ transport in higher plants. *Annals of Botany*.

[11] Munns R, Tester M (2008). Mechanisms of salinity tolerance. *Annual Review of Plant Biology*.

[12] Blumwald E, Aharon GS, Apse MP (2000). Sodium transport in plant cells. *Biochimica et Biophysica Acta: Biomembranes*.

[13] Shi H, Quintero FJ, Pardo JM, Zhu J (2002). The putative plasma membrane NA^+^/H^+^ antiporter SOS1 controls long-distance NA+ transport in plants. *The Plant Cell Online*.

[14] Apse MP, Aharon GS, Snedden WA, Blumwald E (1999). Salt tolerance conferred by overexpression of a vacuolar Na^+^/H^+^ antiport in Arabidopsis. *Science*.

[15] Shi H, Lee B, Wu S, Zhu J (2003). Overexpression of a plasma membrane Na^+^/H^+^ antiporter gene improves salt tolerance in *Arabidopsis thaliana*. *Nature Biotechnology*.

[16] Zhang H, Hodson JN, Williams JP, Blumwald E (2001). Engineering salt-tolerant Brassica plants: characterization of yield and seed oil quality in transgenic plants with increased vacuolar sodium accumulation. *Proceedings of the National Academy of Sciences of the United States of America*.

[17] Guan B, Hu Y, Zeng Y, Wang Y, Zhang F (2011). Molecular characterization and functional analysis of a vacuolar Na^+^/H^+^ antiporter gene (HcNHX1) from *Halostachys caspica*. *Molecular Biology Reports*.

[18] Jha A, Joshi M, Yadav NS, Agarwal PK, Jha B (2011). Cloning and characterization of the Salicornia brachiata Na^+^/H^+^ antiporter gene SbNHX1 and its expression by abiotic stress. *Molecular Biology Reports*.

[19] Rauf M, Shahzad K, Ali R (2014). Cloning and characterization of Na^+^/H^+^ antiporter (LfNHX1) gene from a halophyte grass Leptochloa fusca for drought and salt tolerance. *Molecular Biology Reports*.

[20] Feki K, Quintero FJ, Khoudi H (2014). A constitutively active form of a durum wheat Na^+^/H^+^ antiporter SOS1 confers high salt tolerance to transgenic Arabidopsis. *Plant Cell Reports*.

[21] Xing J, Wang B, Jia K (2011). Isolation of Arachis hypogaea Na^+^/H^+^ antiporter and its expression analysis under salt stress. *African Journal of Biotechnology*.

[22] Wang S, Li Z, Rui R, Fan GS, Lin KW (2013). Cloning and characterization of a plasma membrane Na^+^/H^+^ antiporter gene from Cucumis sativus. *Russian Journal of Plant Physiology*.

[23] Liu Q, Xu K, Zhong M (2013). Cloning and characterization of a novel vacuolar Na^+^/H^+^ antiporter gene (Dgnhx1) from chrysanthemum. *PloS One*.

[24] Blanchard OJ (2008). Innovations in Hibiscus and Kosteletzkya (Malvaceae, Hibisceae). *Novon: A Journal for Botanical Nomenclature*.

[25] Gallagher JL (1985). Halophytic crops for cultivation at seawater salinity. *Plant and Soil*.

[26] Zhou G, Xia Y, Ma BL, Feng C, Qin P (2010). Culture of seashore mallow under different salinity levels using plastic nutrient-rich matrices and transplantation. *Agronomy Journal*.

[27] Gallagher J (1995). Biotechnology approaches for improving halophytic crops: somaclonal variation and genetic transformation. *Biology of Salt-Tolerant Plants*.

[28] Garciadeblás B, Haro R, Benito B (2007). Cloning of two SOS1 transporters from the seagrass *Cymodocea nodosa*. SOS1 transporters from *Cymodocea* and *Arabidopsis* mediate potassium uptake in bacteria. *Plant Molecular Biology*.

[29] Martínez-Atienza J, Jiang X, Garciadeblas B (2007). Conservation of the salt overly sensitive pathway in rice. *Plant Physiology*.

[30] Shi H, Ishitani M, Kim C, Zhu J (2000). The Arabidopsis thaliana salt tolerance gene SOS1 encodes a putative Na^+^/H^+^ antiporter. *Proceedings of the National Academy of Sciences of the United States of America*.

