# Supplementary material for: Molecular Cloning and Bioinformatics Analysis of a New Plasma Membrane Na+/H+ Antiporter Gene from the Halophyte Kosteletzkya virginica
Source: ScientificWorldJournal. 2014 Jun 30;2014:141675. doi: 10.1155/2014/141675 (PMC4100297; doi:10.1155/2014/141675)
Supplement: Supplementary file 1 — Supplementary Figure 1: The ORF nucleotide sequence and the deduced peptide sequence of KvSOS1. Start codon and termination codon highlighted in red. [file 141675.f1.pdf]

1 M E E L K E N L Y V L P S R I L Q E I S S A S S E P V D A V

1 ATGGAGGAACTGAAGGAGAATCTGTACGTCTTACCCTCGCGAATTTTGCAAGAGATTAGCTCTGCTTCATCGGAGCCCGTTGATGCGGTT

31 I F V G I S L A L G I A C R H I F R G T K V P Y T V A L L I

91 ATCTTCGTTGGGATTTCTCTGGCATTAGGAATTGCTTGCCGACATATTTTCCGCGGTACCAAAGTTCCCTACACTGTCGCTTTGCTTATC

61 I G I G L G S I E Y G T S H K L G R I G D G I R I W N N I D

181 ATTGGCATCGGTCTCGGCTCTATTGAATATGGTACAAGTCATAAATTAGGAAGGATTGGAGATGGTATTCGTATTTGGAACAACATTGAT

91 P D L LL A V F L P A L L F E S A F S M E V H Q I K R C M A

271 CCTGACCTTCTATTAGCTGTTTTCCTACCTGCTTTACTTTTTGAGAGTGCATTTTCTATGGAAGTGCACCAGATAAAGAGGTGTATGGCA

121 Q M V L L A G P G V L I S T F C L G S A L K L T F P Y K W D

361 CAAATGGTTCTACTTGCTGGTCCTGGAGTTCTTATTTCGACCTTCTGCCTTGGATCTGCTCTGAAGCTCACTTTTCCATACAAGTGGGAC

151 W K T S L LL G G L L S A T D P V A V V A L L K D L G A S K

451 TGGAAAACATCGCTGTTGCTTGGGGGACTTCTTAGTGCCACTGATCCTGTTGCTGTTGTGGCATTATTGAAGGATCTTGGTGCTAGCAAA

181 K L S T I I E G E S L M N D G T A I V V Y Q L F Y K M V L G

541 AAACTGAGCACCATAATTGAAGGCGAATCCTTGATGAATGATGGGACAGCAATTGTGGTCTATCAGTTATTCTATAAAATGGTACTTGGA

211 K S F S W D A I I E F L A K V S L G A V A V G I A F G I A S

631 AAGAGCTTTAGTTGGGATGCCATTATTGAATTTCTGGCCAAAGTCTCACTTGGAGCCGTGGCGGTTGGAATTGCTTTTGGGATAGCATCA

241 V L W L G F I F N D T V I E I T L T L A V S Y I V Y F T A Q

721 GTTTTGTGGCTCGGATTTATTTTCAATGATACAGTGATTGAGATTACATTGACACTTGCTGTGAGCTACATTGTTTACTTCACTGCTCAA

271 E G I E V S G V L A V M T L G M F Y A A V A K T A F K G D S

811 GAAGGTATTGAAGTTTCCGGTGTTTTGGCAGTGATGACATTAGGAATGTTTTATGCGGCTGTTGCAAAGACAGCCTTTAAGGGTGATAGC

301 Q Q S L H H F W E M V A Y I A N T L I F I L S G V V I A E G

901 CAGCAGAGCTTGCACCACTTTTGGGAAATGGTTGCCTATATTGCAAATACATTAATTTTCATCCTGAGTGGGGTTGTTATAGCTGAGGGC

331 I L G D D K I F H N N E H S W G Y L I L L Y I F I Q V S R C

991 ATTCTTGGCGATGATAAGATATTTCATAACAATGAACATTCTTGGGGCTATCTGATTCTTTTGTACATCTTTATCCAAGTATCACGCTGC

361 I V V G A L Y P F L R Y L G Y G L D L K E A V I L I W S G L

1081 ATTGTTGTTGGAGCATTATATCCATTTTTACGATATCTTGGATATGGTTTGGATTTAAAGGAAGCCGTCATCCTAATATGGTCAGGGCTT

391 R G A V A L S L S L S V K R S S D G S Q Y I S S E T G S L F

1171 CGAGGGGCTGTTGCATTATCACTTTCTCTATCTGTTAAGCGTTCCAGTGACGGCTCACAATATATCAGTTCTGAAACAGGAAGCCTGTTT

421 I F F T G G I V F L T L I V N G S T T Q F V L H F L D M D K

1261 ATTTTCTTCACTGGTGGAATTGTATTCTTGACACTTATTGTGAACGGATCAACTACACAGTTCGTTTTACATTTTCTGGATATGGATAAA

451 L S A A K K R I L D Y T K Y E M L N K A F E A F E D L G D D

1351 CTATCAGCAGCCAAGAAGCGTATTCTGGACTACACAAAGTATGAAATGTTGAACAAAGCATTCGAGGCTTTTGAAGACCTTGGAGATGAT

481 E E L G P A D W P T V K R Y I A S L N D L E G D P V H P H T

1441 GAGGAACTTGGACCTGCTGATTGGCCCACGGTAAAGAGATACATTGCAAGCTTAAACGATTTGGAGGGGGACCCTGTGCATCCTCACACT

511 E S E A D N N L D P S N L K D I R V R L L N G V Q S S Y W G

1531 GAATCTGAAGCTGATAATAATCTGGACCCTTCAAATTTGAAAGATATACGAGTACGGCTTTTAAATGGTGTCCAGTCATCATACTGGGGA

541 M L D E G R I S Q T T A N L L M Q S V D E A I D V A S H E P

1621 ATGCTTGATGAAGGGAGAATTTCACAAACTACAGCAAATCTATTGATGCAATCTGTAGATGAAGCTATTGATGTGGCATCTCATGAACCT

571 L C D W K G L K S N V H F P N Y Y K F L Q S S M F P Q K L I

1711 TTATGTGATTGGAAGGGCTTAAAATCTAATGTTCATTTCCCAAATTATTACAAGTTTCTTCAGTCAAGTATGTTCCCTCAAAAACTGATT

601 T Y F T V E R L E N A C C V C A A F L R A H R I A R R Q L H

1801 ACGTATTTCACTGTGGAAAGGCTGGAAAATGCATGCTGTGTTTGTGCTGCATTTCTTCGAGCTCATAGAATTGCACGACGGCAGCTTCAT

631 E F I G D S V V A S T V I S E S E A E G E E A R K F L E D V

1891 GAGTTTATAGGTGACAGTGTTGTTGCTTCTACTGTAATTTCTGAAAGTGAGGCTGAAGGAGAAGAGGCAAGGAAGTTTTTGGAAGATGTC

661 R I T F P Q V L R V V K T R Q V T Y S V L N H L I E Y L Q N

1981 CGTATAACTTTTCCGCAGGTTTTGCGTGTTGTTAAGACAAGACAAGTTACCTACTCGGTATTGAACCATCTGATTGAGTATTTACAAAAC

691 L E K V G L L E E K E M L H L H D A V Q T D L K K L L R N P

2071 CTCGAGAAGGTTGGGTTACTGGAAGAAAAAGAAATGCTTCATCTTCATGATGCTGTCCAGACTGACTTGAAGAAGCTTTTAAGGAATCCT

721 P L V K I P K I N D L I S A H P L L G A L P S T I C E K L L

2161 CCCTTGGTAAAGATTCCGAAGATAAATGATCTAATAAGTGCCCATCCTTTGCTAGGCGCCCTTCCTTCTACTATCTGCGAGAAACTATTA

751 G Y T K E K M K T R G M T L Y K E G S K S N G I W L V S N G

2251 GGTTATACAAAAGAAAAAATGAAAACTCGTGGTATGACACTTTACAAAGAGGGCTCTAAATCAAATGGTATTTGGCTAGTTTCAAACGGT

781 V V K W T S R S I R N K H S V H P T F S H G S T L G L Y E V

2341 GTTGTCAAGTGGACGAGTAGGAGCATAAGAAACAAGCATTCAGTGCATCCAACTTTTAGTCATGGGAGTACGTTGGGCTTGTACGAAGTA

811 L V G K P Y I C D M V T D S V V L C I F I E S D R I L S V L

2431 TTGGTTGGAAAACCATACATCTGCGACATGGTCACAGATTCCGTGGTTCTCTGTATTTTTATTGAGAGTGACAGAATACTTTCAGTACTA

841 R S D P D I E D F L W R E S A L V L A K L L V P Q I F E K M

2521 AGGTCGGATCCTGACATAGAAGATTTTCTCTGGCGGGAAAGTGCTCTTGTCCTCGCCAAACTCTTGGTTCCTCAAATATTTGAGAAAATG

871 A L H D L R A L V A E R S S M K T Y I A G E T I E V S H Q L

2611 GCACTGCACGATTTAAGAGCTCTTGTAGCAGAAAGGTCGTCGATGAAGACATACATTGCAGGGGAAACAATAGAAGTGTCACACCAATTG

901 I G F L L E G F A K P L L A Q E E L I T S P A V L L P S Q G

2701 ATTGGCTTCTTGTTGGAAGGGTTCGCGAAACCTTTACTTGCTCAAGAAGAACTCATCACATCACCAGCAGTTCTTTTGCCTTCACAAGGG

931 N Q S F L Y A D K S G S A T T S F S H Q R S G Y Q L E T R G

2791 AATCAAAGTTTCTTATATGCAGATAAATCAGGTTCTGCAACAACCAGCTTTTCTCATCAGCGATCTGGGTATCAACTTGAGACAAGAGGA

961 S I I Y Q V E T R A R A I I F D I A T L E A N R V L R R N S

2881 AGCATAATATATCAAGTTGAGACTAGAGCAAGAGCAATTATTTTTGATATTGCAACACTTGAAGCCAATAGAGTTTTGCGGAGAAATTCC

991 S S F T H S H K S L I R E H G G L M S W P E N F F S G R Q H

2971 TCCTCATTTACCCATTCACACAAAAGTTTAATTAGAGAACATGGGGGTCTTATGAGTTGGCCTGAAAACTTCTTCAGCGGAAGACAACAT

1021 T Q N H E E S D Q Q V N S L S A R A M Q L S I F G S T V D L

3061 ACACAAAATCATGAAGAAAGTGATCAACAAGTAAACAGCTTATCTGCAAGAGCCATGCAGTTAAGCATCTTTGGCAGCACGGTTGATTTG

1051 P R R S R S L S R M H Q S K P A Y N R S Y D R I L S F P G H

3151 CCACGGCGCAGCCGGAGTTTATCAAGGATGCATCAATCTAAACCAGCATACAACCGGTCATATGATAGAATTCTTTCATTCCCTGGACAT

1081 P L V S G R S E G S V T M R K N L E E G R K I T R P L P P A

3241 CCACTGGTTTCTGGCAGATCAGAAGGATCTGTTACAATGAGGAAGAATCTCGAAGAAGGGAGAAAGATAACAAGACCGTTACCCCCCGCA

1111 Q A K N T D S K E G H G N D E S D E D E I L V R I D S P S G

3331 CAAGCGAAGAACACGGACTCGAAAGAGGGCCACGGAAATGATGAAAGTGATGAAGATGAAATCCTGGTGAGGATCGATTCGCCTAGCGGA

1141 L S F N Q A S *

3421 CTATCATTCAACCAAGCTTCTTGA

Supplementary Figure 1: The ORF nucleotide sequence and the deduced peptide sequence of

KvSOS1. Start codon and termination codon highlighted in red.
